# Result of the Use of Chloroform in 9000 Cases at Bartholomew’s Hospital

**Published:** 1851-07

**Authors:** 


*Result of the Use of Chloroform in* 9000 *cases at Bartholomew's Hospital.* By Mr. Skey.

One of the most interesting questions connected with the subject of operative surgery relates to the use of anesthetic agents employed for the purpose of suspending the function of sensation. This question has assumed a moral as well as a medical type. It has been urged, that sensation is a natural function of the living organism, and that to suspend it by artificial agency, is to set at nought the ordinances of nature; and that man is born to suffering, as evidenced by the sensibilities of his body. If the soundness of this argument be admitted, it would be difficult to draw a line which would define the boundary at which moral and *immoral* suffering meet; or to say, in what form of suffering our remedial agents may be justifiably resorted to. The sensibilities of our frame are not given us by nature to the end of promoting pain, but to enable us to avoid it. Corporal suffering is no part of the discipline of the mind; nor can it even be generally asserted that its excess exercises a salutary influence on the character. Every movement of our body instinctively points to the avoidance of bodily suffering; why, therefore, should we not as readily and unobjectionably employ the agency of anesthetic medicines for the purpose of suspending bodily pain, under the circumstances of an otherwise painful operation, as we endeavor to mitigate the bodily suffering of any other patient cast down on a bed of sickness? Will not the objection to the anesthetic action of opium to a region affected by a neuralgic pain, or to the system generally, hold as strongly as that of another agent of the same principle given to avert the pain of an operation?

The medical arguments against the use of anesthetic agents have a somewhat better foundation. That great and sudden determination to the brain, and an unnatural circulation of venous blood, result from their employment, is undeniable.

It is undeniable, if the quantity administered be large, and long continued, that symptoms resembling those of apoplexy present themselves, in the form of extreme congestion of the vessels of the face, stertorous respiration, and total insensibility; and it cannot be denied, that occasionally its full administration leads to headache, vertigo, and languor of some days duration; and cases are recorded in which death itself has followed in the course of an hour or more after its employment. It must be observed, however, in pursuing this question in strict accordance with the laws of evidence, that we have no *proof*, in the cases above referred to, that death was the direct effect of the supposed cause. The parties administering it were not fully experienced in the mode of its application. They entertain the *opinion* that death was referable to it, while it cannot be disputed that the fatal issue may be attributable to other causes; and, in one example, it appears more reasonable to refer the death of the individual to a suspension of the function of respiration by violence, than to any obnoxious agent circulating through the lungs, or brain. On the other hand, the records of St. Bartholomew's Hospital point to its successful administration in upwards of 9000 cases; in not one of which, including the aged and the young, the healthy, the infirm and the asthmatic, has its employment left a stain on its character, as an innocuous agent of good. Under all circumstances, its careful employment may be unhesitatingly resorted to in all cases, excepting only such as are marked by determination to the brain of an apoplectic type; secondly, under circumstances of great and serious exhaustion from loss of blood; and, thirdly, in diseases of the heart. In these conditions of the system, it is perhaps better avoided.

The agent in general use is chloroform, and one word may be added as to its administration. It appears indisputable, that its influence on sensation precedes that on consciousness. I have employed it on several occasions, in which a patient has been conscious of all that has been passing around, and yet who has declared himself to have been totally insensible to pain. This state of his system has arisen from the moderate use of the agent, ample, indeed, for all purposes of utility, though somewhat difficult to regulate in quantity sufficient for the required object.

I prefer its gradual administration. I do not think it desirable to exclude atmospheric air, employed as a diluent during the process of inhalation. Its influence should be gradual, not sudden, I consider its application through the medium of a cambric handkerchief laid on the face, preferable to the use of instruments made for the purpose of excluding atmospheric air, and food should be rigidly avoided before its administration, otherwise sickness will frequently follow.

Against the occasional convictions or objections of others to its employment, I place the strong, and to my own mind the unanswerable fact, that it has been successfully used in so large a number of cases in St. Bartholomew's Hospital, since the period of its introduction; that these cases have been indiscriminately taken, and that its objections have not yet made their appearance before the observant eyes of the medical staff of that institution, either by promoting danger during the operation, or protracting the recovery of the patient after it. In one class of cases its employment is especially applicable, viz., in that form of disease in which the pain of an operation is the chief warrant for its nonperformance, and in which the recovery from a chronic disease is left to nature, that might be greatly hastened by the hand of art; such, for example, as the removal of a piece of dead bone.

Up to the period of the introduction of chloroform, a surgeon was very unwilling to subject a patient to the painful process of sawing and chipping away portions of dead bone, with a view to reach the medullary cavity, because the operation was both a painful and protracted one. The consequence was, that an hospital bed was occupied by a patient thus affected, for many months, to the exclusion, perhaps, of three or more claimants, who would have successively occupied it. But by the aid of chloroform the operation is now performed unconsciously to the patient, and the period of his recovery greatly abridged. With the three exceptions above mentioned, I cannot hesitate in strongly recommending its administration in all cases of large surgical operations; believing its discovery to be the greatest blessing conferred on the profession of surgery during the last century; and although I have seen its employment pushed, on many occasions, apparently to the verge of apoplexy, I cannot say, even in such examples, that the good has not largely predominated.—*Operative Surgery.*

